# Discriminating pseudoprogression and true progression in diffuse infiltrating glioma using multi-parametric MRI data through deep learning

**DOI:** 10.1038/s41598-020-77389-0

**Published:** 2020-11-23

**Authors:** Joonsang Lee, Nicholas Wang, Sevcan Turk, Shariq Mohammed, Remy Lobo, John Kim, Eric Liao, Sandra Camelo-Piragua, Michelle Kim, Larry Junck, Jayapalli Bapuraj, Ashok Srinivasan, Arvind Rao

**Affiliations:** 1 Department of Computational Medicine and Bioinformatics, University of Michigan, Ann Arbor, MI USA; 2 Department of Radiology, University of Michigan, Ann Arbor, MI USA; 3 Department of Pathology, University of Michigan, Ann Arbor, MI USA; 4 Department of Radiation Oncology, University of Michigan, Ann Arbor, MI USA; 5 Department of Neurology, University of Michigan, Ann Arbor, MI USA

**Keywords:** Neuroscience, Cancer, Head and neck cancer

## Abstract

Differentiating pseudoprogression from true tumor progression has become a significant challenge in follow-up of diffuse infiltrating gliomas, particularly high grade, which leads to a potential treatment delay for patients with early glioma recurrence. In this study, we proposed to use a multiparametric MRI data as a sequence input for the convolutional neural network with the recurrent neural network based deep learning structure to discriminate between pseudoprogression and true tumor progression. In this study, 43 biopsy-proven patient data identified as diffuse infiltrating glioma patients whose disease progressed/recurred were used. The dataset consists of five original MRI sequences; pre-contrast T1-weighted, post-contrast T1-weighted, T2-weighted, FLAIR, and ADC images as well as two engineered sequences; T1post–T1pre and T2–FLAIR. Next, we used three CNN-LSTM models with a different set of sequences as input sequences to pass through CNN-LSTM layers. We performed threefold cross-validation in the training dataset and generated the boxplot, accuracy, and ROC curve, AUC from each trained model with the test dataset to evaluate models. The mean accuracy for VGG16 models ranged from 0.44 to 0.60 and the mean AUC ranged from 0.47 to 0.59. For CNN-LSTM model, the mean accuracy ranged from 0.62 to 0.75 and the mean AUC ranged from 0.64 to 0.81. The performance of the proposed CNN-LSTM with multiparametric sequence data was found to outperform the popular convolutional CNN with a single MRI sequence. In conclusion, incorporating all available MRI sequences into a sequence input for a CNN-LSTM model improved diagnostic performance for discriminating between pseudoprogression and true tumor progression.

## Introduction

Diffuse infiltrating gliomas can be astrocytic or oligodendroglial in nature. Astrocytomas are graded from low grade (WHO grade 2) to anaplastic (WHO grade 3) and glioblastoma (WHO grade 4). Whereas oligodendroglial tumors can present as low grade (WHO grade 2) or anaplastic (WHO grade 3). Regardless, progression is part of the natural disease, and determining true tumor progressive disease (PD) versus pseudoprogression (PsP) or treatment related changes is important for appropriate management and clinical decision making. More than half of these gliomas are glioblastomas (GBM)^1^ in which the median survival for GBM patients is about 15 months^2,3^ despite the radiation and chemotherapy treatment and the average 2-year survival rate is only 8–12%^4^. The current standard treatment for gliomas is surgical resection of the tumor followed by radiation therapy and chemotherapy, which are used to slow down the growth of residual tumor after surgery^5^.

MRI is the mainstay in the assessment of the tumor in the preoperative and postoperative period. During treatment and follow-up, in MRI studies, the treated tumors show an increase in size and enhancement in the absence of tumor recurrence without clinical deterioration. This is due to treatment-related effects and this can be divided into pseudoprogression (PsP) or radiation necrosis^6^. In this study, we defined PsP according to the Response Assessment in Neuro-Oncology (RANO) criteria as follow; PsP is radiologically defined as new or enlarging area of contrast enhancement occurring early after the completion of radiotherapy in the absence of true PD^7–10^. PsP is thought to represent a localized tissue reaction with an inflammatory component and associated edema and abnormal vascular permeability which explains the imaging features^11^. This imaging features is similar to true tumor progression. Distinguishing PsP from true progressive disease (PD) is an imaging challenge especially in the cerebral white matter^11^. The temporal profile of appearance is crucial. PsP typically occurs early in the post treatment period; in approximately 60% of cases it occurs within the first 3 months of completion of treatment but the duration varies from a weeks to up to 6 months after treatment^12–14^. It was recently reported that the incidence of PsP ranges from 9 to 30%^8,10,15–17^. In contrast, radiation necrosis may appear months to several years after radiation therapy and involves a space occupying necrotic lesion with mass effect and neurological dysfunction^6,18^.

Differentiating PsP from true PD has become a significant challenge in high grade glioma follow-up, which leads to a potential treatment delay for patients with early tumor recurrence^6^. The current standard practice for diagnosis of recurrent disease is surgical biopsy but it is an invasive procedure with limited in accuracy depending on the biopsy site and lesion heterogeneity^12,19^. Alternatively, various conventional and advanced MRI sequences such as pre- and post-contrast T1-weighted images, T2-weighted images, and fluid attenuated inversion recovery (FLAIR) have been used to differentiate PsP from true PD with follow-up scans^10^. The diagnosis of PsP on these MR images is based on the changes in the lesion site on follow-up images. However, this usually takes several weeks to identify PsP and true PD.

Several studies have investigated in differentiating PsP from PD using advanced MRI techniques. Galldiks et al. assessed the clinical value of *O*-(2-^18^F-fluoroethyl)-l-tyro-sine (^18^F-FET) PET in the differentiation of PsP and early tumor progression after radiochemotherapy of glioblastoma. They investigated a group of 22 glioblastoma patients with new contrast-enhancing lesions on standard MRI within the first 12 weeks after completion of radiochemotherapy with concomitant temozolomide. They showed that ^18^F-FET PET could be a promising method for overcoming the limitations of conventional MRI in differentiating PsP from early tumor progression^20^. Hu et al. investigated relative cerebral blood (rCBV) volume values to differentiate high-grade glioma recurrence from post-treatment radiation effect using localized dynamic susceptibility-weighted contrast-enhanced perfusion MR imaging (DSC) measurements. They used forty tissue specimens collected from 13 subjects and found a threshold value of 0.71 differentiating the histopathologic groups with a sensitivity of 91.7% and a specificity of 100%. They showed that rCBV measurements obtained by using DSC can differentiate high-grade glioma recurrence from the tumor recurrence and post-treatment radiation effect. Prager et al. and Wang et al. investigated the utility of diffusion and perfusion imaging to differentiate tumor progression from pseudoprogression^21,22^. Chuang et al. examined roles of several metabolites in differentiating recurrent tumor from necrosis in patients with brain tumor using MR perfusion^23^. Detsky et al. investigated that IVIM-based diffusion and perfusion measurements would be able to differentiate post-radiation recurrent or progressive tumor from radiation necrosis^24^. Reimer et al. investigated whether a voxel-wise analysis of ADC values may differentiate between PD and PsP^25^.

Attempts in differentiating PsP from PD have included the use of several computer-aided diagnosis systems such as texture analysis^26^, radiomics^27^, machine learning^28,29^, and deep learning^30,31^. Image texture analysis has been used to characterize the spatial distribution and assess the repeating patterns of local variations in gray-level intensities within an image^32^, providing important information on tumor microenvironment^33^. Chen et al. uses second-order statistics, such as contrast, energy, entropy, correlation, and homogeneity, from gray level co-occurrence matrix (GLCM) to differentiate PsP and true PD on MRI images^26^. Kim et al. developed a radiomics model using multiparametric MRI to differentiate PsP from early tumor progression in patients with glioblastoma^27^. Several studies showed that the artificial intelligence and machine learning methods are useful in solving diagnostic decision-making problems in clinical research. Hu et al. uses machine learning algorithms with multiparametric MRI features to identify PsP from tumor recurrence in patients with resected Glioblastoma^28^. Recently, Jang et al. investigated the feasibility of a combination of a convolutional neural network (CNN) and a long short-term memory (LSTM) deep learning model to determine PsP and true PD in GBM patients^30^. Their dataset consisted of nine successive axial images of post-contrast T1-weighted sequence before and after administration of gadolinium. Their CNN-long short term memory (CNN-LSTM) structure with clinical and MRI data yielded high performance in differentiating PsP from true PD in GBM patients (an area under the curve [AUC] of 0.83)^30^.

The primary application of CNNs is classifying images and performing object recognition within an image. Recurrent neural networks (RNNs), on the other hand, are superior for learning temporal patterns in deep neural networks. RNN applications encompass a variety of problems such as speech recognition, language translation, and image captioning. LSTM is a special type of RNN, which can remember each and every information through time. The combination of CNN and LSTM is capable of learning a series of time-based images in a specific MRI modality. In this study, instead of using a series of time-based images, we proposed a set of multiparametric MRI data as a spatial sequence input for the CNN-LSTM framework, which can remember each and every information through multiparametric MR images. Our proposed approach uses all available MRI sequences at a specific time point and can incorporate all information from each sequence and predict patients’ outcome. As far as we know, this is the first study for the proposed CNN-LSTM with multiparametric MRI data as a spatial sequence input.

To evaluate the performance of the CNN-LSTM model with our proposed sequential data, we estimated the area under the ROC curve (AUC) and compared that with the fine-tuned VGG16 network, which is one of the most popular deep learning models that was pre-trained on the ImageNet dataset^34^. The hypothesis for this study is that our proposed CNN-LSTM model with all available MRI sequence data will show better diagnostic performance than the conventional CNN model with a single MRI sequence as input for discriminating between PsP and true PD in diffuse infiltrating gliomas.

## Results

**Figure 1:**
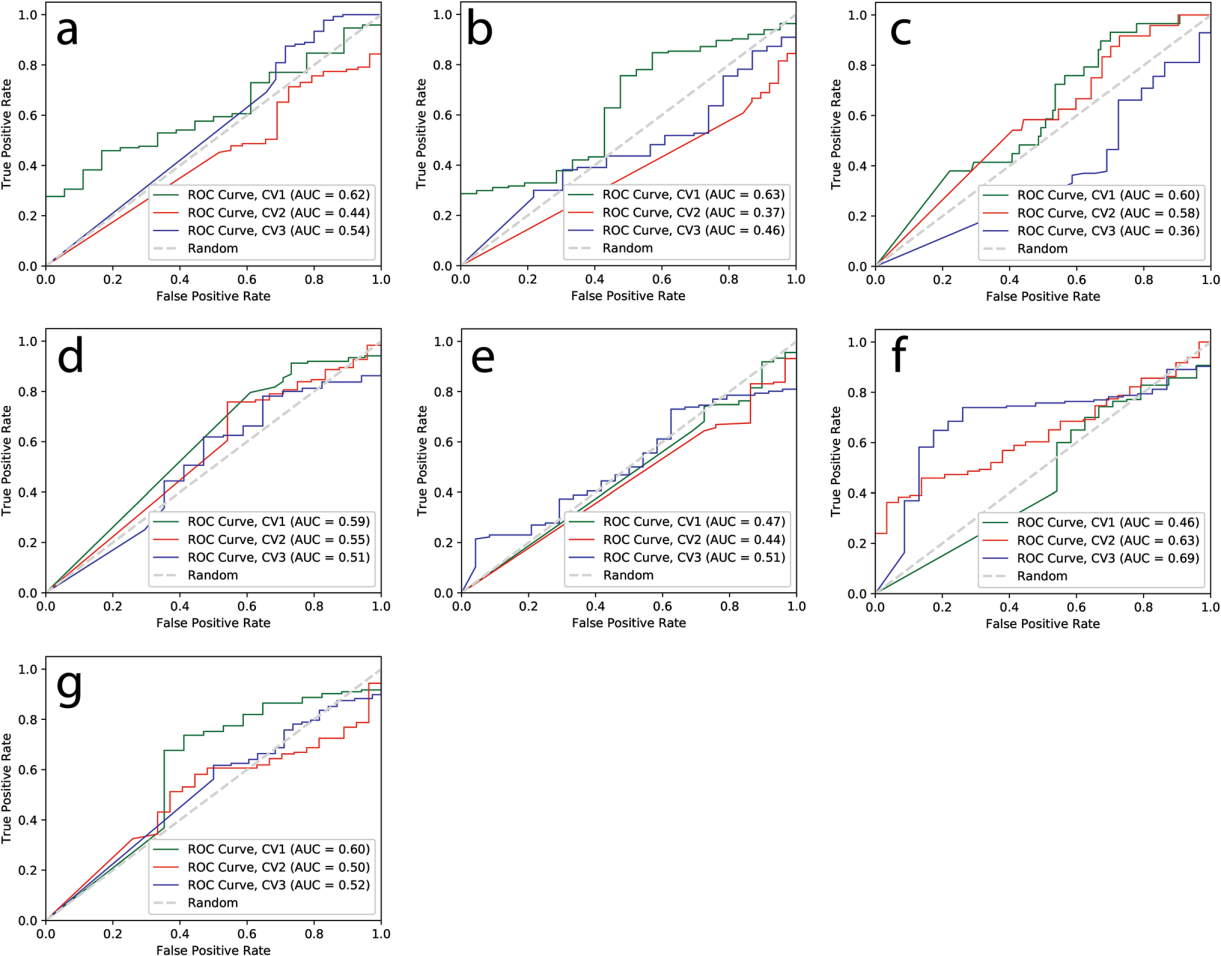
ROC curves for VGG16 deep learning models with each MRI sequence for prediction of PsP from TP. Each ROC curve for each modality obtained from threefold cross-validation (CV1, CV2, and CV3). The x-axis is the true negative rate (TNR) or specificity and the y-axis is true positive rate (TPR) or sensitivity. The mean AUC, area under ROC curve, values were (**a**) 0.53 for pre T1-weighted, (**b**) 0.49 for post T1-weighted, (**c**) 0.51 for T2-weighted, (**d**) 0.55 for FLAIR, (**e**) 0.47 for ADC map, (**f**) 0.59 for post T1–pre T1, (**g**) 0.54 for T2–FLAIR images.

For VGG16 model, we split the dataset of each modality into three folds and fed it into VGG16 architecture to build a model for classification problem of PsP. Table 1 summarize the results for the classification accuracy and AUC with 95% confidence interval for each modality for VGG16 models. The mean accuracy for all modalities in VGG16 models ranged from 0.44 to 0.60 and total mean accuracy for the VGG16 model is 0.52. The mean AUC for all sequences ranged from 0.47 to 0.59 and total mean AUC for VGG16 model is 0.53. For the CNN-LSTM model, we prepared a set of dataset with multiple modalities as a spatial sequence dataset; 3, 5, and 7 MRI sequences. The mean accuracy for each spatial sequence dataset is 0.62, 0.70, and 0.75, respectively and the mean AUC for each spatial sequence dataset is 0.64, 0.69, and 0.81, respectively (Table 2, Fig. 1). These mean accuracies and AUC values were computed from threefold cross-validation for both VGG16 and CNN-LSTM models.

**Table 1:** Classification mean accuracy, AUC, and 95% C.I. for VGG16 models.

	Accuracy	AUC	95% C.I
T1 pre	0.51	0.53	[0.47–0.64]
T1 post	0.48	0.49	[0.39–0.59]
T2	0.44	0.51	[0.41–0.62]
FLAIR	0.55	0.55	[0.44–0.67]
ADC	0.48	0.47	[0.38–0.57]
T1post–T1pre	0.60	0.59	[0.49–0.70]
T2–FLAIR	0.58	0.54	[0.42–0.66]

**Table 2:** Classification mean accuracy, AUC, and 95% C.I. for CNN-LSTM models.

	Accuracy	AUC	95% C.I
3 modalities: T1 pre, T1 post, and T2	0.62	0.64	[0.51–0.77]
5 modalities: T1 pre, T1 post, T2, FLAIR, and ADC	0.70	0.69	[0.59–0.79]
7 modalities: T1 post–T1 pre, T2–FLAIR, and 5 modalities	0.75	0.81	[0.72–0.88]

**Figure 2:**
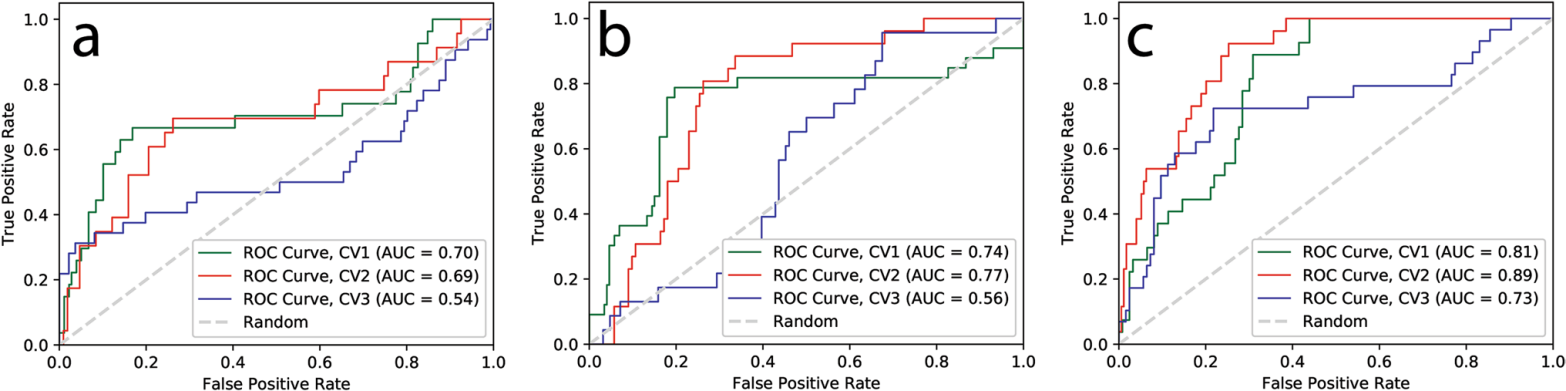
ROC curves for CNN-LSTM deep learning models with a different set of sequences for prediction of PsP from TP. Each ROC curve for each modality obtained from threefold cross-validation (CV1, CV2, and CV3). The x-axis is the true negative rate (TNR) or specificity and the y-axis is true positive rate (TPR) or sensitivity. The mean AUC, area under ROC curve, values were (**a**) 0.64 for a set of 3 modalities, (**b**) 0.69 for a set of 5 modalities, and (**c**) 0.81 for a set of 7 modalities.

**Figure 3:**
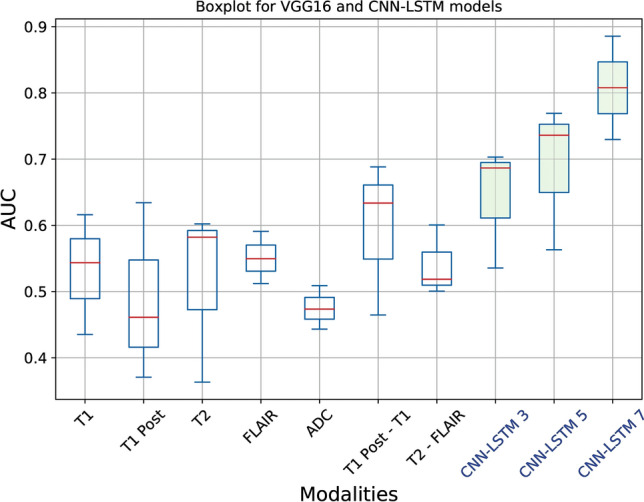
Boxplots of AUC for VGG16 and CNN-LSTM models. The first seven box plots are for VGG16 with an individual MRI sequence, and last three box plots are for CNN-LSTM with multiparametric MRI sequences. Statistics were collected from threefold cross validation and the red lines in the boxplots represent the median values.

We applied threefold cross-validation in the training set and boxplots were computed form this threefold validation. A set of 3, 5, and 7 multiparametric MRI data for CNN-LSTM model have the mean AUC of 0.64, 0.69, and 0.81, respectively (Fig. 2). Figure 3 shows boxplots of AUC for VGG16 and CNN-LSTM models. For the results with threefold cross-validation, please see Tables S1–S4 in the Supporting information.

## Discussion

Following the extensive revisions in the 2016 WHO classification of CNS tumors, there has been further iteration of the classification giving a more granular definition of gliomas. The term diffuse gliomas is the more preferred term for tumors with tumors based on astrocytic, oligodendroglia or a mixture of both with tumor behavior further determined by their molecular fingerprint based on IDH mutations and presence of the 1p19q co-deletions. The concept of diffuse gliomas has been further enhanced by more recent recommendations^35,36^. Regardless of the classifications, the treatment of these tumors is challenge, given the recent reports of the median survival for glioblastoma patients at about 15 months and a median progression free survival of 6.2–7.5 months^37^. During the treatment, an increasing number of patients show changes in tumor enhancement patterns in MRI in absence of true PD. This becomes a major challenge in glioblastoma follow-up and leads to a potential treatment delay for patients with early glioblastoma recurrence. Recently, the classification problem of PsP and true PD based on machine learning with multiparametric MR images has received increasing interest. Tiwari et al. investigated distinguishing brain tumor progression from pseudoprogression on routine MRI using machine learning^38^. Abrol et al. investigated differencing PsP from true PD in glioblastoma patients using radiomic analysis^39^. However, only a few studies proposed differentiating PsP from true PD based on the deep neural network framework^30,40,41^. The potential underlying reasoning for this is insufficient data, inability of deep learning to capture real variations in the data unless the data size is large enough. Deep learning requires large amounts of data more than any other machine learning algorithm. This is a typical problem in healthcare.

LSTM is a special kind of recurrent neural network that is designed to avoid the long-term dependency problems and vanishing gradient problems that are typically encountered in RNNs. In recent years, LSTMs have received increasing attention in the medical domain such as predicting future medical outcomes^42^, segmentation of pancreas in MRI images^43^, automated diagnosis of arrhythmia with variable length heart beats^44^ and classification of histopathological breast cancer images^45^.

Jang et al. applied the CNN-LSTM structure to determine PsP from true PD in GBM patients. Their dataset consisted of MRI data and clinical feature data with 78 patients (48 PsP and 30 true PD patients)^30^. They incorporated clinical feature data into MRI data and their CNN-LSTM structure with both clinical and MRI data outperformed the model of CNN-LSTM with MRI data alone (AUC = 0.69).

The typical limitation of deep neural networks in clinical applications is the relative lack of data, making them vulnerable to inaccuracies^46^. For this reason, our proposed approach with a multiparametric MRI sequence dataset will overcome this limitation and have the benefit of using all available imaging sequences in a MRI study. Further, this method has the capability of learning important information over the multiparametric MRI sequence dataset simultaneously.

In the present study, we included two modalities, T1post–T1pre and T2–FLAIR, by subtracting pre-contrast T1-weighted images from post-contrast T1-weighted images and FLAIR images from T2-weighted images, respectively^47^. These two engineered modalities will give us the extent of the enhancing areas and indirectly indicates the more fluid tissue against solid tissue, respectively. In theory, the FLAIR sequence is similar to a T2-weighted image but solid abnormalities remain bright but fluid tissues become dark. The subtraction of T2-weighted from FLAIR gives us information about more fluid tissue against solid tissue. Our proposed CNN-LSTM can incorporate all information from each sequence and predict patients’ outcome. In addition, the rationale of subtracting the pre- and post-contrast T1 weighted sequence was to accurately segment the enhancing portion of the lesion. The enhancing solid portions of the tumor on the T1 weighted image may exhibit heterogeneous T2 hyperintense signal. Perifocal edema is also hyperintense on the T2 and FLAIR sequence. The idea of subtracting the T2 and FLAIR sequence is to demonstrate 1) the enhancing solid T2 heterogeneous portion of the lesion and separating it from non-solid non-enhancing edema. Assessment of the T2 hyperintense portions of the lesion is particularly important on the basis of the new concerns of this appearance on GBMs as noted in the recent papers^48^.

In this study, we examined the feasibility of CNN-LSTM with a set of multiparametric MRI sequence data for identifying PsP from true PD. There are limitations in our retrospective study. First, it appeared that the VGG16 model with each modality does not have a good classification ability. This is because we still have a relatively small dataset for PsP (n = 7) and true PD (n = 36) for each modality. However, our proposed approach adds a multifaceted analysis by combining all MRI modalities when compared with the case of analyzing individual modalities. As a result, the proposed model confers a relatively high AUC of 0.81 under the best CNN-LSTM model. Future studies could validate these results using a larger sample size. Second, our results for CNN-LSTM models show increasing performance when we use more multiparametric MRI data as spatial sequence data. However, it appeared that the computational time for training increases according to the number of modalities in a set of sequence data. For example, a set of 3-modalities sequence data used 50 epochs to reach the best results while a set of 5-modalities sequence data and a set of 7-modalities sequence data used 200 and 500 epochs to reach the best results, respectively. This is not surprising since CNN-LSTM take a set of multiparametric MRI sequence dataset as an input. However, optimizing the CNN-LSTM structure may be required for the best performance and computational time in the future study. In this study, deep learning models were trained on our workstation with an Intel i9 3.50 Ghz 12-core processor, 128 GB system memory, and a single GPU (NVIDIA GeForce RTX 2080 TI).

In this study, we demonstrated the state-of-the-art CNN and RNN-based deep neural network, CNN-LSTM, with our proposed data frame, a set of multiparametric MRI data. These multiparametric MRI data as a spatial sequence input were fed into CNN layers for feature extraction. Then, the output from the CNN layers was fed into the LSTM layer to take all sequences into account for the final prediction. Incorporating all available MRI sequences into a sequence input for a CNN-LSTM model improved diagnostic performance for discriminating between pseudoprogresson and true tumor progression by leveraging correlation between multiple sequences. Therefore, our proposed method with a CNN-LSTM model and multiparametric MRI data combines the advantages of both extracting important features from images with CNN and learning from all sequences simultaneously with LSTM.

We believe that this study is novel in two aspects. First, we increased image data for the deep learning algorithm by creating engineered sequences. Second, we incorporated all available MRI modalities, including engineered modalities for our proposed model. These data augmentations are elegant and straightforward, compared to conventional deep learning augmentation methods (e.g., rotation, scaling, flip, etc.) because each MRI sequence was designed to highlight differences in the signal of various tissues representing unique pathological characteristics of tumors. For example, T1-weighted images are useful for evaluation of anatomic tissue structures and T2-weighted images are useful for high signal tissue, including fluid-containing structures. As a corollary to the histopathologically validated machine learning radiographic biomarkers described in the literature^29^, our methodology appears to be more robust given the fact that it discriminates tumor progression across the spectrum of diffuse infiltrating gliomas rather than of glioblastoma alone.

Our proposed model is capable of taking in multiple MRI sequences and leveraging correlation between them simultaneously to distinguish PsP from true PD. There are several studies with multiparametric MRI input data on CNN. However, they are different from our proposed method. Currently, multiparametric MRI data with the deep learning study are either large volumes of multiparametric MRI input data or based on fusion techniques such as image fusion, feature fusion, and classifier fusion^49^. The key difference between CNN and CNN-LSTM is that CNN-LSTM accepts multiple images as a sequence input and remembers and incorporates all input images simultaneously while CNN process an image at a time. LSTM is commonly used with time series data and there are several studies used CNN-LSTM for the classification tasks in MRI but they used temporal (multiple time points) images in a single modality. As far as we know, this is the first study for the proposed CNN-LSTM with multiparametric MRI data as a spatial sequence input.

In this study, we have demonstrated the feasibility of the CNN-LSTM with a set of multiparametric MRI data as a spatial sequence input. This approach was able to discriminate PsP from true PD and the results from our study showed that our proposed CNN-LSTM with multiparametric MRI data outperforms conventional VGG16, one of the most popular deep learning models commonly used in medical image analysis. VGG16 does not have good classification power with our relatively small dataset, while our proposed method with multiparametric MRI data has relatively high classification power with our given dataset, which would be considered a strength of the proposed method. Further, our approach will potentially help the deep neural network model in clinical applications where the image data are not sufficient for training a model by adding a multifaceted analysis with engineered sequences.

## Methods

### Patient data

A retrospective study of 43 biopsy-proven patient data identified as diffuse infiltrating gliomas (astrocytoma or oligodendroglioma) presenting originally as high grade glioma (WHO grade 3 or 4), who underwent adjuvant chemoradiation therapy after gross total surgical tumor resection and multiple follow up MRI scans between 2010 and 2018 were used in this study. This study was carried out in accordance with relevant guidelines and regulations, and this retrospective analysis of data from MR images was approved and written informed consent was waived by the University of Michigan Institutional Review Boards (IRBMED). The studies were retrieved from the Electronic Medical Record Search Engine (EMERSE) and DataDirect databases of the University^50^. Table S5 lists the primary integrated diagnosis and the secondary evaluation for PsP and PD where all tumors are confirmed pathologically to progress to high grade gliomas (WHO grade 3 or 4).

### Imaging data

**Figure 4:**
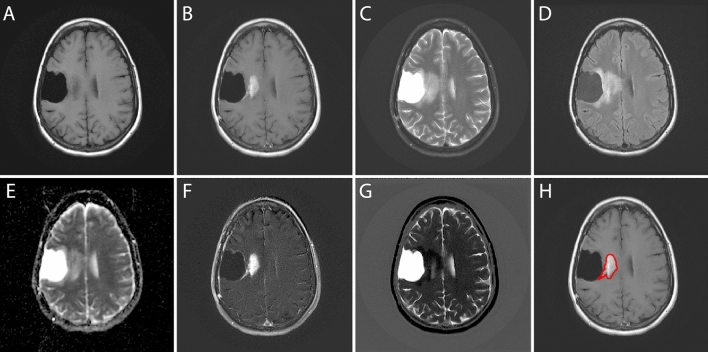
A GBM patient (PsP) with various MRI scans such as (**A**) pre-contrast T1-weighted, (**B**) post-contrast T1-weighted, (**C**) T2-weighted, (**D**) Fluid-Attenuated Inversion Recovery (FLAIR), and (**E**) ADC images. In this study, we included two engineered modalities (**F**) T1post–T1pre and (**G**) T2–FLAIR. And (**H**) the region of interest (ROI) is shown on a post-contrast T1 image.

All MRI studies were obtained following the primary resection procedure. The imaging dataset were selected at three time points. In this study, we only used the MRI data at a time point as a baseline study, which is being closest to the follow-up operation confirming PsP or PD (Table S5). The dataset consists of five different MRI sequences such as pre-contrast T1-weighted, post-contrast T1-weighted, T2-weighted FSE, T2-Fluid-Attenuated Inversion Recovery (FLAIR), and ADC maps. In addition to these, we included two modalities that are generated by subtracting pre-contrast T1-weighted images from post-contrast T1-weighted images (T1post–T1pre) and FLAIR images from T2-weighted images (T2–FLAIR). Figure 4 shows the original five MRI sequences (A–E) and two engineered sequences (F. T1post–T1pre, G. T2–FLAIR) for a patient with PsP. All patients with GBM were imaged using both Philips (Philips Eindhoven, Netherlands) and GE (GE Medical systems, Milwaukee, WI, USA) MR scanners.

All patients had histological proof for recurrence and true PD. Out of 43 patients, seven patients were confirmed by histopathological evaluation to have PsP. The rest 36 patients were confirmed to be true PD cases. Cases diagnosed as pseudo progression (PsP) by histopathology were those cases in which the majority (> 90%) of the resected tissue has histologic features of treatment effect. If a viable residual tumor was seen (< 10%), this has to be minimal or non-proliferative, indicating that the radiologic changes were mostly due to reactive/necrotic tissue rather than proliferative tumor. All MRI images were normalized using white-stripe normalization^51^ in R (R Foundation for Statistical Computing, Vienna, Austria), using the White Stripe package.

### Deep learning structure

Convolutional Neural Network (CNN) has become dominant in various computer vision tasks and attracting interest across a variety of domains including medical image classification tasks. In this study, we used VGG16 and CNN-LSTM architectures. VGG16 is one of the most popular CNN models that was pre-trained on the ImageNet dataset^34^ and was used with various medical image classification tasks. First, we used the VGG16 with each MRI sequence to train model for fine-tuning. All images were resized to 224 × 224 pixels with 3 channels as input. We used the VGG16 model that was pre-trained from ImageNet and this model only accepts input image size of 224 × 224 × 3. We simply stack each MRI grayscale image (224 × 224) to make 3-channel image (224 × 224 × 3) to mimic the RGB structure. This technique is commonly used with a pre-trained deep learning model in medical image analysis^52,53^. VGG16 contains 16 convolutional layers with small receptive fields 3 × 3 and five max-pooling layers with the size of 2 × 2 and involve 144 million parameters. We froze the weights of layers except the weights from last four layers of the VGG16. This step fine-tuned our model with our dataset.

We combined the CNN with RNN based LSTM for the second portion of our study. RNN is specifically designed to take a series of inputs and remembers the previous information. LSTM network is a special type of RNN. LSTM units include a memory cell that can maintain the previous information in the memory cell for a longer period of time. LSTM is typically used in the time series data. All RNN models have the form of a chain of repeating modules of neural network. A common LSTM also has this structure but the LSTM unit is composed of a cell and three gates, namely an input gate, an output gate, and a forget gate. The cell remembers information over the sequence intervals and these gates allow the information to pass to another time step when it is relevant^54^. In this study, we used a combination of CNN and LSTM structures (CNN-LSTM), with multiparametric MR images as a spatial sequence dataset. In this study, we coin the term “spatial sequence” to refer to a set of multiple MRI images for a patient (e.g., the same axial images of T1, T2, and FLAIR sequences) in no specific order to differentiate from the terms commonly used in the MRI sequence (e.g., T1/T2 sequence) and in the LSTM model (e.g., time sequence).

**Figure 5:**
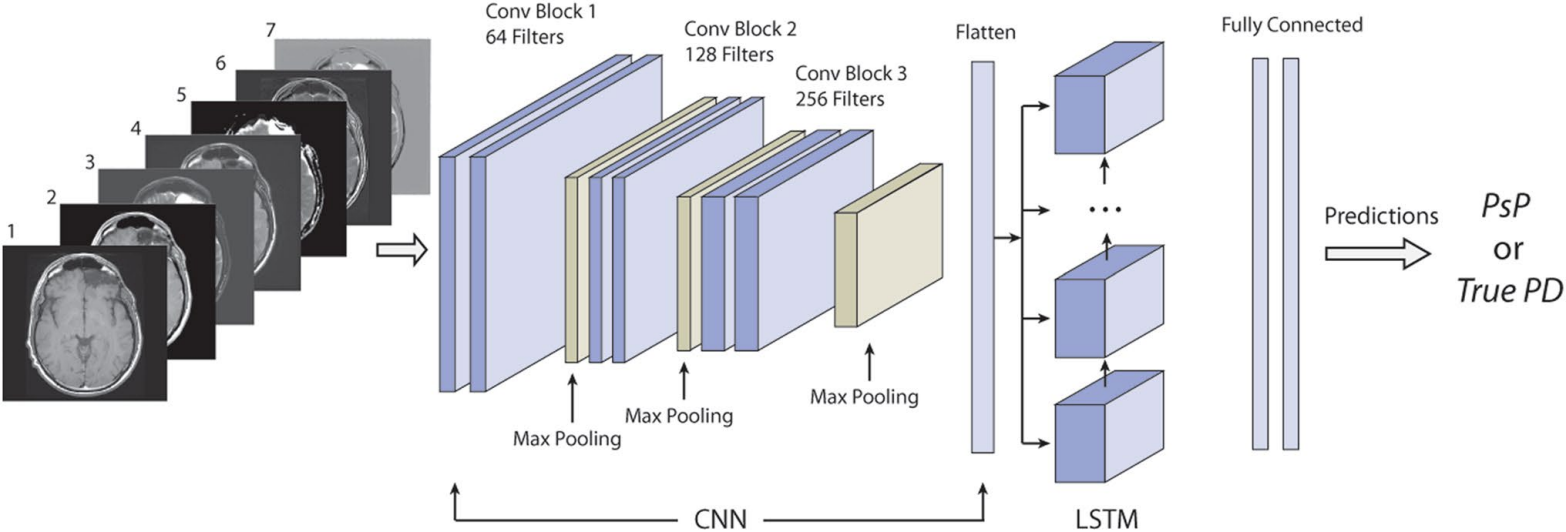
CNN-LSTM architecture with a set of multiparametric MRI sequence input consisting of (1) T1pre, (2) T1post, (3) T2, (4) FLAIR, (5) ADC map, (6) T1post–T1pre, and (7) T2–FLAIR.

First, we used three CNN-LSTM models with a different set of sequences, which are a set of 3 MRI sequences, 5 MRI sequences, and 7 MRI sequences, respectively, as an input spatial sequences to pass through CNN-LSTM layers. In each CNN layer, we used a 2D convolutional layer followed by a max pooling layer with a 2 × 2 kernel size. We added a batch normalization layer to normalize the activations of the previous layer at each batch. This layer maintains the mean activation close to 0 and the activation standard deviation close to 1. Each CNN layer contains 2 × 2 kernels to generate 64, 128, and 256 filters, respectively. We used the binary cross-entropy that measure the performance of a classification model as a loss function and the stochastic gradient descent optimizer to minimize this loss function. The flatten layer was added at the end of the CNN layers to flatten the output and feed into LSTM layers. In LSTM layers, a set of flattened patches from the output of CNN entered LSTM sequentially. The model has a hidden LSTM layer with 24 units followed by a dense layer to provide the output (Fig. 5). CNN-LSTM uses entire images and classify PsP or true PD. The deep learning network structures were implemented in Python, using Keras library with Tensorflow backend (Python 3.6, Keras 2.2.4, Tensorflow 1.12.0).

### Data analysis

In the fine-tuned VGG16 Model, various augmentation methods were applied to the training dataset such as rescaling, rotation, shift, shear, and horizontal flip using image data preprocessing function in the Keras neural network library. MR images for each sequence were fed into the VGG16 model. For the CNN-LSTM model, we created 3 sets of spatial sequence dataset such as a set of 3, 5, and 7 MRI sequences. The detail modalities in each spatial sequence dataset are listed in Table 2. In each spatial sequence dataset, we included two engineered sequences that were generated by subtracting pre-contrast T1-weighted images from post-contrast T1-weighted images to show the extent of the enhancing area of the tumor. Then, we subtracted FLAIR images from T2-weighted images to show the difference between the perifocal edema and the more substantial portion of the tumor.

We performed a threefold cross-validation in the training dataset and generated the confusion matrix, accuracy, and ROC curve from each trained models with the test dataset for each fold. Also, we estimated the area under the ROC curve (AUC) with a 95% confidence interval to evaluate the trained model in the test dataset.

## Supplementary information

Supplementary Information.

## Data Availability

The datasets generated during and/or analyzed during the current study are available from the corresponding author on reasonable request.
