# Supplementary material for: Discriminating pseudoprogression and true progression in diffuse infiltrating glioma using multi-parametric MRI data through deep learning
Source: Sci Rep. 2020 Nov 23;10:20331. doi: 10.1038/s41598-020-77389-0 (PMC7683728; doi:10.1038/s41598-020-77389-0)
Supplement: Supplementary file 1 — Supplementary Information. [file 41598_2020_77389_MOESM1_ESM.docx]

**Discriminating Pseudoprogression and True Progression in Diffuse Infiltrating Glioma using Multi-parametric MRI data through Deep Learning**

Joonsang Lee^1^, Nicholas Wang^1^, Sevcan Turk^2^, Shariq Mohammed^1^, Remy Lobo^2^, John Kim^2^, Eric Liao^2^, Sandra Camelo- Piragua^3^, Michelle Kim^4^, Larry Junck^5^, Jayapalli Bapuraj^2^, Ashok Srinivasan^2^, Arvind Rao^1,^*

^1^Department of Computational Medicine and Bioinformatics, University of Michigan, Ann Arbor, Michigan, United States of America

^2^Department of Radiology, University of Michigan, Ann Arbor, Michigan, United States of America

^3^Department of Pathology, University of Michigan, Ann Arbor, Michigan, United States of America

^4^Department of Radiation Oncology, University of Michigan, Ann Arbor, Michigan, United States of America

^5^Department of Neurology University of Michigan, University of Michigan, Ann Arbor, Michigan, United States of America

*Corresponding Author:

E-mail: ukarvind@med.umich.edu (AR)

**Table S1**. Confusion matrix and classification accuracy for VGG16 models

|  | 3-fold | Confusion matrix | | acc | Acc | Mean acc |
| --- | --- | --- | --- | --- | --- | --- |
|  |  | PsP | PD |  |  |  |
| T1 | 1 | 10 | 8 | 0.56 | 0.55 | 0.51 |
|  |  | 78 | 92 | 0.54 |  |  |
|  | 2 | 13 | 16 | 0.45 | 0.46 |  |
|  |  | 62 | 53 | 0.46 |  |  |
|  | 3 | 12 | 23 | 0.34 | 0.52 |  |
|  |  | 42 | 94 | 0.69 |  |  |
| T1post | 1 | 12 | 9 | 0.57 | 0.60 | 0.48 |
|  |  | 61 | 103 | 0.63 |  |  |
|  | 2 | 6 | 32 | 0.16 | 0.40 |  |
|  |  | 53 | 82 | 0.61 |  |  |
|  | 3 | 10 | 13 | 0.43 | 0.44 |  |
|  |  | 62 | 48 | 0.44 |  |  |
| T2 | 1 | 14 | 15 | 0.48 | 0.49 | 0.44 |
|  |  | 72 | 68 | 0.49 |  |  |
|  | 2 | 11 | 13 | 0.46 | 0.45 |  |
|  |  | 87 | 67 | 0.44 |  |  |
|  | 3 | 11 | 18 | 0.38 | 0.38 |  |
|  |  | 80 | 47 | 0.37 |  |  |
| Flair | 1 | 16 | 25 | 0.39 | 0.60 | 0.55 |
|  |  | 28 | 109 | 0.80 |  |  |
|  | 2 | 11 | 13 | 0.46 | 0.53 |  |
|  |  | 49 | 75 | 0.60 |  |  |
|  | 3 | 9 | 8 | 0.53 | 0.52 |  |
|  |  | 79 | 81 | 0.51 |  |  |
| ADC | 1 | 9 | 20 | 0.31 | 0.48 | 0.48 |
|  |  | 48 | 87 | 0.64 |  |  |
|  | 2 | 8 | 21 | 0.28 | 0.46 |  |
|  |  | 57 | 103 | 0.64 |  |  |
|  | 3 | 12 | 12 | 0.50 | 0.50 |  |
|  |  | 63 | 63 | 0.50 |  |  |
| T1post  -T1 | 1 | 11 | 13 | 0.46 | 0.46 | 0.60 |
|  |  | 77 | 63 | 0.45 |  |  |
|  | 2 | 17 | 12 | 0.59 | 0.59 |  |
|  |  | 60 | 86 | 0.59 |  |  |
|  | 3 | 17 | 6 | 0.74 | 0.74 |  |
|  |  | 43 | 122 | 0.74 |  |  |
| T2 - Flair | 1 | 11 | 6 | 0.65 | 0.67 | 0.58 |
|  |  | 43 | 90 | 0.68 |  |  |
|  | 2 | 15 | 12 | 0.56 | 0.55 |  |
|  |  | 75 | 85 | 0.53 |  |  |
|  | 3 | 19 | 19 | 0.50 | 0.53 |  |
|  |  | 56 | 72 | 0.56 |  |  |
| **Total mean ACC** | |  | | | | **0.52** |

**Table S2**. Confusion matrix and classification accuracy for CNN-LSTM models

|  | 3-fold | Confusion matrix | | ACC | Mean ACC | Average |
| --- | --- | --- | --- | --- | --- | --- |
|  |  | PsP | PD |  |  |  |
| A set of 3 modalities | 1 | 18 | 9 | 0.67 | 0.64 | 0.62 |
|  |  | 72 | 106 | 0.60 |  |  |
|  | 2 | 16 | 7 | 0.70 | 0.72 |  |
|  |  | 28 | 79 | 0.74 |  |  |
|  | 3 | 16 | 16 | 0.50 | 0.50 |  |
|  |  | 69 | 67 | 0.49 |  |  |
| A set of 5 modalities | 1 | 26 | 7 | 0.79 | 0.80 | 0.70 |
|  |  | 34 | 139 | 0.80 |  |  |
|  | 2 | 19 | 7 | 0.73 | 0.74 |  |
|  |  | 31 | 91 | 0.75 |  |  |
|  | 3 | 13 | 10 | 0.57 | 0.57 |  |
|  |  | 55 | 71 | 0.56 |  |  |
| A set of 7 modalities | 1 | 18 | 9 | 0.67 | 0.70 | 0.75 |
|  |  | 35 | 88 | 0.72 |  |  |
|  | 2 | 21 | 5 | 0.81 | 0.81 |  |
|  |  | 35 | 139 | 0.80 |  |  |
|  | 3 | 21 | 8 | 0.72 | 0.75 |  |
|  |  | 27 | 97 | 0.78 |  |  |

**Table S3**. AUC and 95% C.I for VGG16

|  | 3-fold | AUC | 95% C.I. | mean AUC |
| --- | --- | --- | --- | --- |
| T1 | 1 | 0.62 | [ 0.51 – 0.72] | 0.57 |
|  | 2 | 0.56 | [ 0.46 – 0.66] |  |
|  | 3 | 0.54 | [ 0.45 – 0.64] |  |
| T1 post | 1 | 0.63 | [ 0.51 – 0.75] | 0.49 |
|  | 2 | 0.37 | [ 0.31 – 0.44] |  |
|  | 3 | 0.46 | [ 0.35 – 0.58] |  |
| T2 | 1 | 0.60 | [ 0.49 – 0.71] | 0.51 |
|  | 2 | 0.58 | [ 0.48 – 0.68] |  |
|  | 3 | 0.36 | [ 0.26 – 0.47] |  |
| FLAIR | 1 | 0.59 | [ 0.51 – 0.68] | 0.55 |
|  | 2 | 0.55 | [ 0.44 – 0.67] |  |
|  | 3 | 0.51 | [ 0.36 – 0.65] |  |
| ADC | 1 | 0.47 | [ 0.38 – 0.60] | 0.47 |
|  | 2 | 0.44 | [ 0.36 – 0.53] |  |
|  | 3 | 0.51 | [ 0.40 – 0.58] |  |
| T1p – T1 | 1 | 0.47 | [ 0.34 – 0.59] | 0.60 |
|  | 2 | 0.63 | [ 0.54 – 0.72] |  |
|  | 3 | 0.69 | [ 0.58 – 0.78] |  |
| T2 - FLAIR | 1 | 0.60 | [ 0.45 – 0.75] | 0.54 |
|  | 2 | 0.50 | [ 0.40 – 0.61] |  |
|  | 3 | 0.52 | [ 0.42 – 0.61] |  |
| **Total mean AUC** | |  | | **0.53** |

**Table S4**. AUC and 95% C.I for CNN-LSTM

|  | 3-fold | AUC | 95% C.I. | Mean AUC |
| --- | --- | --- | --- | --- |
| A set of 3 modalities | 1 | 0.70 | [0.57 – 0.83] | 0.64 |
|  | 2 | 0.69 | [0.55 – 0.81] |  |
|  | 3 | 0.53 | [0.40 – 0.67] |  |
| A set of 5 modalities | 1 | 0.74 | [0.62 – 0.85] | 0.69 |
|  | 2 | 0.77 | [0.68 – 0.85] |  |
|  | 3 | 0.56 | [0.46 – 0.66] |  |
| A set of 7 modalities | 1 | 0.81 | [0.73 – 0.87] | 0.81 |
|  | 2 | 0.89 | [0.83 – 0.93] |  |
|  | 3 | 0.73 | [0.62 – 0.84] |  |

**Table S5**. Patient Data

|  | **Sex** | **Date Original Resection** | **Original Integrated Diagnosis** | **Date 2^nd^ Resection** | **Diagnosis of 2^nd^ Resection PsP & PD** | **Age TP1** | **TP 1  (baseline study)** | **TP 2** | **TP 3** |
| --- | --- | --- | --- | --- | --- | --- | --- | --- | --- |
| 1 | M | 10/20/2008 | Oligodendroglioma, IDH-mutant, WHO grade 2 | 01/19/2012 | PD | 31 | 17-Dec-12 | 18-Sep-12 | 26-Jun-12 |
| 2 | M | 06/03/2015 | Glioblastoma, IDH-wildtype, WHO grade 4 | 05/09/2017 | PD | 66 | 11-Jul-17 | 20-Apr-17 | 7-Dec-16 |
| 3 | M | 2004 | Diffuse Astrocytoma, NOS | 01/09/2015 | PD | 62 | 5-Jan-15 | 24-Oct-13 | 11-Oct-12 |
| 4 | M | 01/25/2011 | Oligodendroglioma, IDH-mutant, WHO grade 2 | 12/15/2016 | PD | 53 | 4-Oct-16 | 22-Jan-16 | 21-May-15 |
| 5 | M | 07/09/2015 | Glioblastoma, IDH-wildtype, WHO grade 4 | 09/23/2016 | PsP | 58 | 3-Aug-16 | 13-Nov-15 | 17-Aug-15 |
| 6 | M | 04/29/2011 | Oligodendroglioma, IDH-mutant, WHO grade 2 | 01/11/2017 | PD | 51 | 22-Dec-16 | 3-Oct-16 | 27-Jul-16 |
| 7 | M | 1998 | Diffuse Astrocytoma, IDH-mutant, NOS | 08/07/2014 | PD | 62 | 6-Jul-14 | 7-May-14 | 5-Mar-14 |
| 8 | F | 04/23/2014 | Glioblastoma, IDH-wildtype, WHO grade 4 | 02/19/2015 | PD | 59 | 25-Jan-15 | 29-Dec-14 | 27-Oct-14 |
| 9 | F | 4/25/2017 | Glioblastoma, IDH-wildtype, WHO grade 4 | 1/3/2018 | PsP | 70 | 20-Nov-17 | 28-Sep-17 | 3-Aug-17 |
| 10 | M | 2000 | Oligodendroglioma,IDH-wildtype, WHO grade 2 | 12/1/2016 | PD | 38 | 15-Sep-16 | 28-Feb-15 | 15-Sep-13 |
| 11 | F | 04/08/2014 | Glioblastoma, IDH-wildtype, WHO grade 4 | 6/25/2015 | PD | 67 | 12-May-15 | 13-Mar-15 | 11-Jan-15 |
| 12 | M | 02/08/2006 | Diffuse Astrocytoma, WHO grade 2 NOS | 03/07/2014 | PD | 31 | 28-Feb-14 | 17-Jan-14 | 25-Oct-13 |
| 13 | M | 1997 | Oligodendroglioma, IDH-mutant, WHO grade 2 | 7/10/2014 | PD | 65 | 3-Jul-14 | 14-May-14 | 22-Jan-14 |
| 14 | F | 07/25/2012 | Astrocytoma , IDH-mutant, WHO grade 2 | 06/19/2015 | PD | 35 | 20-Apr-15 | 19-Jan-15 | 20-Oct-14 |
| 15 | M | 02/10/2003 | Oligodendroglioma, IDH-mutant, WHO grade 2 | 01/22/2015 | PsP | 46 | 23-Dec-14 | 25-Oct-14 | 19-Jul-14 |
| 16 | F | 12/19/2008 | Oligodendroglioma, IDH-mutant, WHO grade 2 | 10/18/2016 | PD | 55 | 12-Sep-16 | 9-Oct-14 | 18-Mar-09 |
| 17 | M | 2/23/2015 | Glioblastoma, WHO grade 4 NOS | 10/04/2017 | PD | 45 | 19-Sep-17 | 27-Jul-17 | 8-Jun-17 |
| 18 | M | 06/11/2001 | Oligodendroglioma, WHO grade 2 | 02/08/2017 | PD | 53 | 1-Dec-16 | 8-Jun-16 | 9-Dec-15 |
| 19 | M | 3/24/15 | Glioblastoma, IDH-wildtype, WHO grade 4 | 05/31/2016 | PD | 55 | 9-May-16 | 14-Mar-16 | 13-Feb-16 |
| 20 | M | 12/12/2012 | Glioblastoma, WHO grade 4 NOS | 09/15/2015 | PD | 57 | 14-Aug-15 | 26-Jun-15 | 27-Mar-15 |
| 21 | F | 06/06/2012 | Glioblastoma, WHO grade 4 NOS | 07/18/2013 | PD | 51 | 1-Jul-13 | 29-Apr-13 | 11-Mar-13 |
| 22 | M | 2017 | Astrocyatoma, IDH-wildtype, at least WHO grade 3 | 07/3/2018 | PD | 67 | 30-Jun-18 | 29-May-18 | 27-Feb-18 |
| 23 | F | 2007 | Oligodendroglioma, IDH-mutant, WHO grade 2 | 02/20/2012 | PD | 54 | 17-Feb-12 | 17-Jan-12 | 22-Nov-11 |
| 24 | F | 5/31/12 | Glioblastoma, WHO grade 4 NOS | 10/01/2013 | PD | 57 | 21-Aug-13 | 18-Jun-13 | 18-Apr-13 |
| 25 | F | 01/14/2000 | Oligodendroglioma, IDH-mutant, WHO grade 2 | 04/26/2016 | PD | 52 | 23-Feb-16 | 24-Sep-15 | 14-Nov-14 |
| 26 | F | 6/4/2007 | Oligodendroglioma, IDH-mutant, WHO grade 2 | 12/01/2016 | PD | 52 | 25-Oct-16 | 30-Apr-16 | 10-Nov-15 |
| 27 | M | 11/15/2002 | Oligodendroglioma, IDH-mutant, WHO grade 2 | 03/25/2015 | PD | 51 | 9-Mar-15 | 1-Dec-14 | 28-May-14 |
| 28 | M | 5/4/2016 | Glioblastoma, IDH-wildtype, WHO grade 4 | 05/16/2018 | PD | 55 | 1-May-18 | 6-Mar-18 | 3-Nov-17 |
| 29 | F | 1/31/2013 | Glioblastoma, IDH-mutant, WHO grade 4 | 9/18/2015 | PD | 40 | 22-Aug-15 | 23-May-15 | 18-Apr-15 |
| 30 | F | 6/17/2014 | Oligodendroglioma, IDH-mutant, WHO grade 2 | 05/05/2016 | PsP | 39 | 18-Apr-16 | 11-Jan-16 | 12-Oct-15 |
| 31 | M | 06/12/2014 | Glioblastoma, IDH-wildtype, WHO grade 4 | 01/14/2015 | PD | 52 | 17-Dec-14 | 10-Oct-14 | 5-Aug-14 |
| 32 | M | 08/01/2008 | Glioblastoma, WHO grade 4 NOS | 01/11/2012 | PD | 16 | 9-Jan-12 | 21-Dec-11 | 18-Jul-11 |
| 33 | F | 07/29/2015 | Glioblastoma, WHO grade 4 NOS | 05/11/2016 | PD | 74 | 24-Apr-16 | 14-Mar-16 | 18-Jan-16 |
| 34 | M | 12/16/2011 | Glioblastoma, WHO grade 4 NOS | 07/08/2014 | PD | 56 | 2-Jun-14 | 7-Apr-14 | 10-Feb-14 |
| 35 | F | 1/8/2009 | Astrocytoma , WHO grade 2 NOS | 2/9/2015 | PD | 32 | 2-Jan-15 | 18-Sep-14 | 15-Apr-14 |
| 36 | F | 4/19/2013 | Glioblastoma, IDH-wildtype, WHO grade 4 | 1/12/2016 | PD | 67 | 13-Dec-15 | 2-Sep-15 | 8-Jan-14 |
| 37 | M | 4/11/2014 | Glioblastoma, WHO grade 4 NOS | 2/23/2016 | PD | 64 | 8-Jan-16 | 11-Dec-15 | 9-Oct-15 |
| 38 | F | 2/29/2016 | Glioblastoma, IDH-wildtype, WHO grade 4 | 7/11/2016 | PD | 68 | 4-Oct-16 | 7-Sep-16 | 12-Jul-16 |
| 39 | F | 07/10/2014 | Anaplastic Astrocytoma, IDH-wildtype, WHO grade 3 | 08/10/2016 | PD | 58 | 3-Aug-16 | 31-Jul-16 | 14-Apr-16 |
| 40 | F | 01/12/2011 | Glioblastoma, IDH-wildtype, WHO grade 4 | 06/19/2015 | PD | 51 | 1-Nov-12 | 12-Jul-12 | 12-Apr-12 |
| 41 | M | 9/17/2014 | Glioblastoma, IDH-wildtype, WHO grade 4 | 7/20/2018 | PsP | 37 | 19-Jul-18 | 14-Jun-18 | 13-Mar-18 |
| 42 | F | 1/9/2017 | Glioblastoma, IDH-wildtype, WHO grade 4 | 9/15/2017 | PsP | 67 | 15-Jan-18 | 20-Nov-17 | 31-Jul-17 |
| 43 | M | 10/25/2012 | Glioblastoma, IDH-mutant, WHO grade 4 | 6/20/2013 | PsP | 51 | 10-Jun-13 | 24-Apr-13 | 6-Mar-13 |
